# Supplementary figures and images for: Identification of an Autophagy-Related Signature for Prognosis and Immunotherapy Response Prediction in Ovarian Cancer
Source: Biomolecules. 2023 Feb 9;13(2):339. doi: 10.3390/biom13020339 (PMC9953331; doi:10.3390/biom13020339)

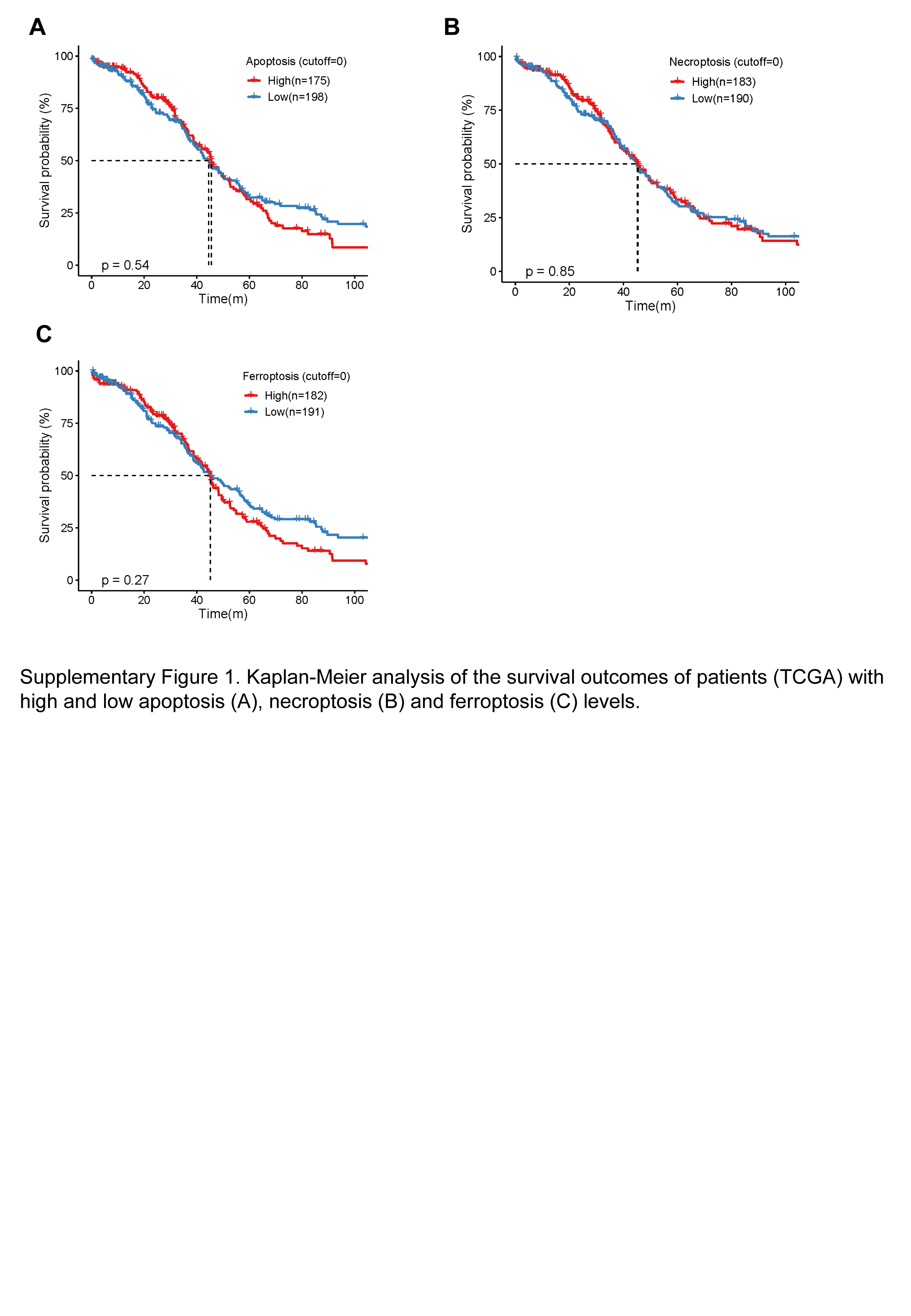

Supplement: Supplementary file 1 [file biomolecules-13-00339-s001.zip › Figure S1.tif]

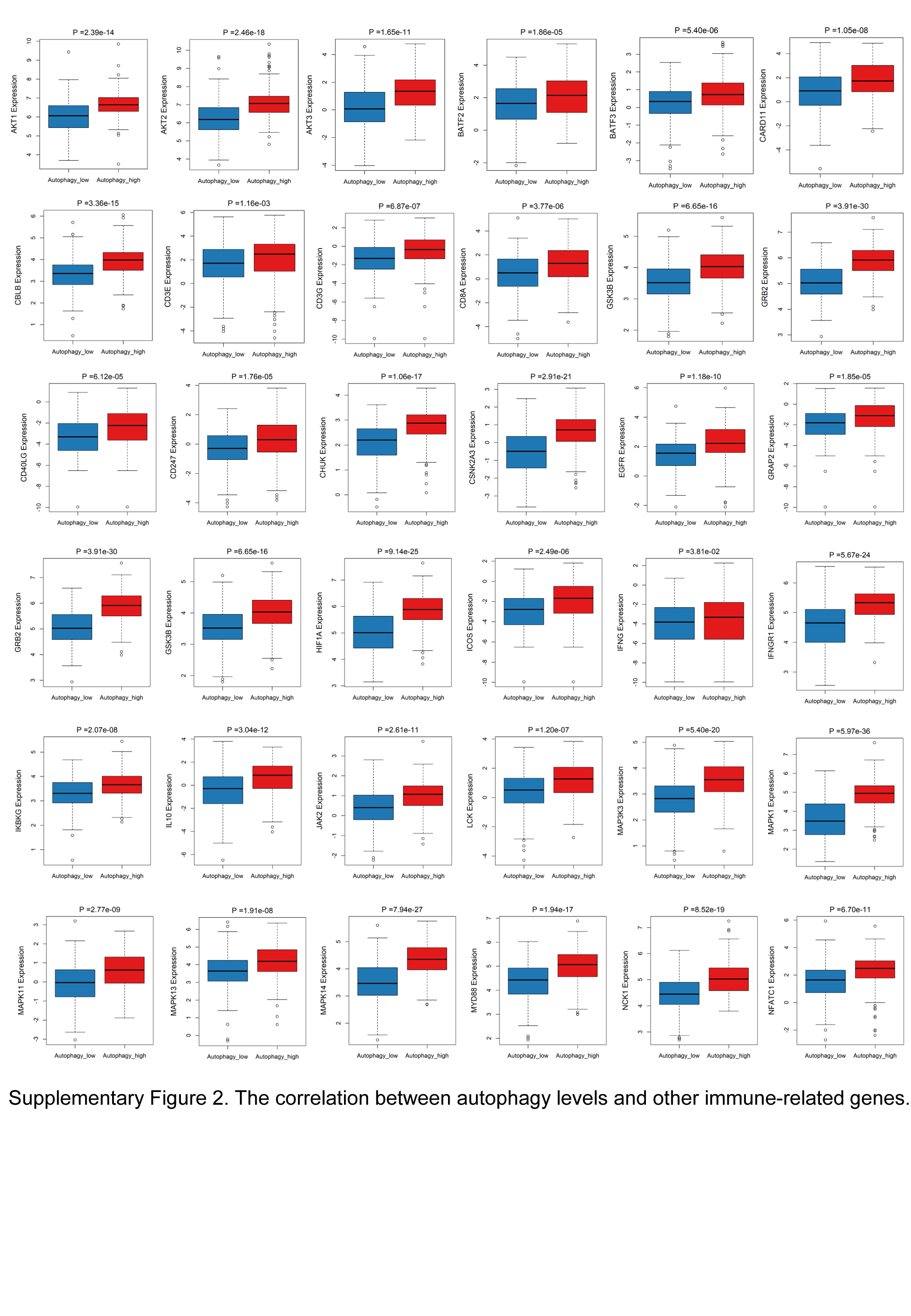

Supplement: Supplementary file 1 [file biomolecules-13-00339-s001.zip › Figure S2-1.tif]

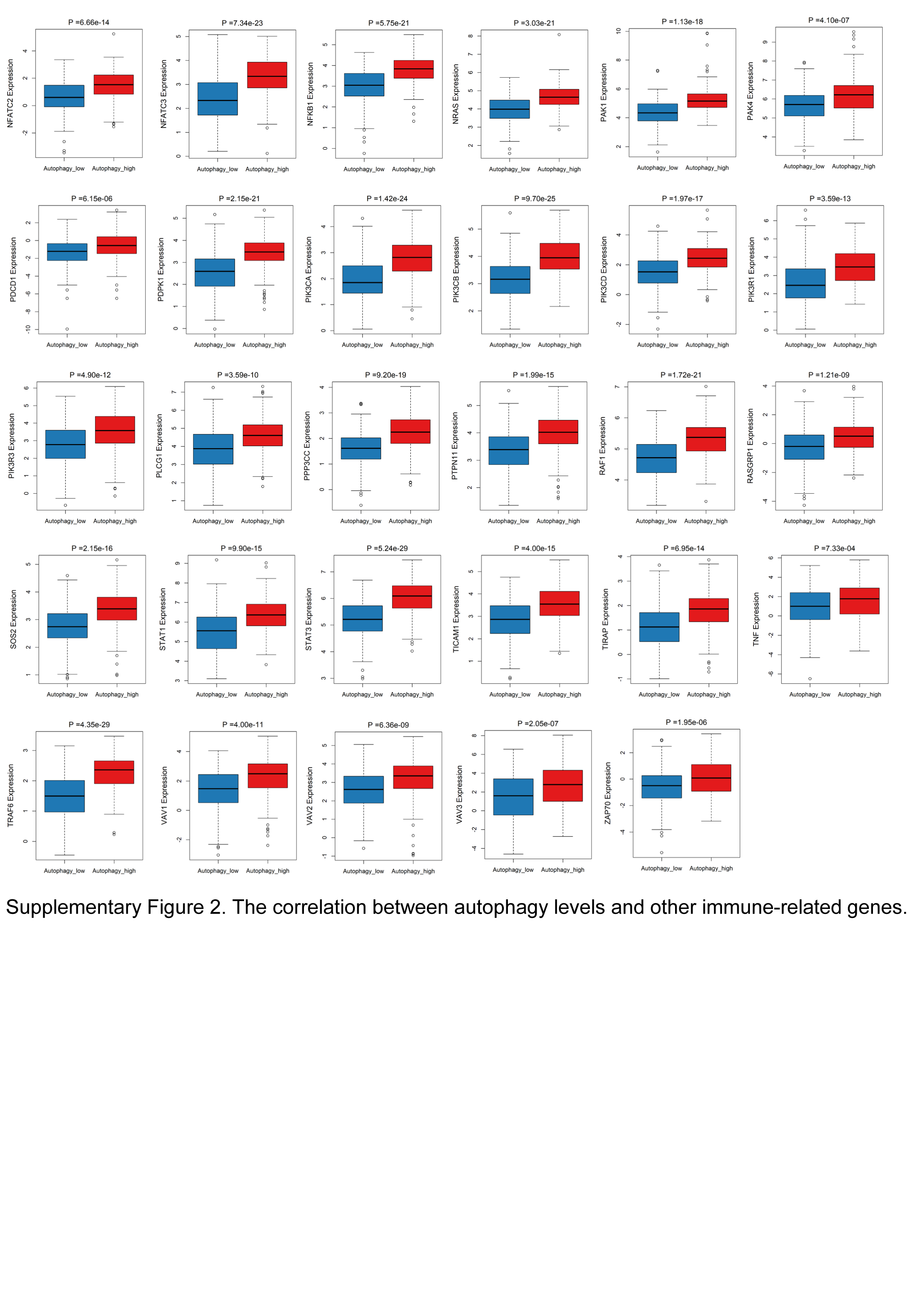

Supplement: Supplementary file 1 [file biomolecules-13-00339-s001.zip › Figure S2-2.tif]

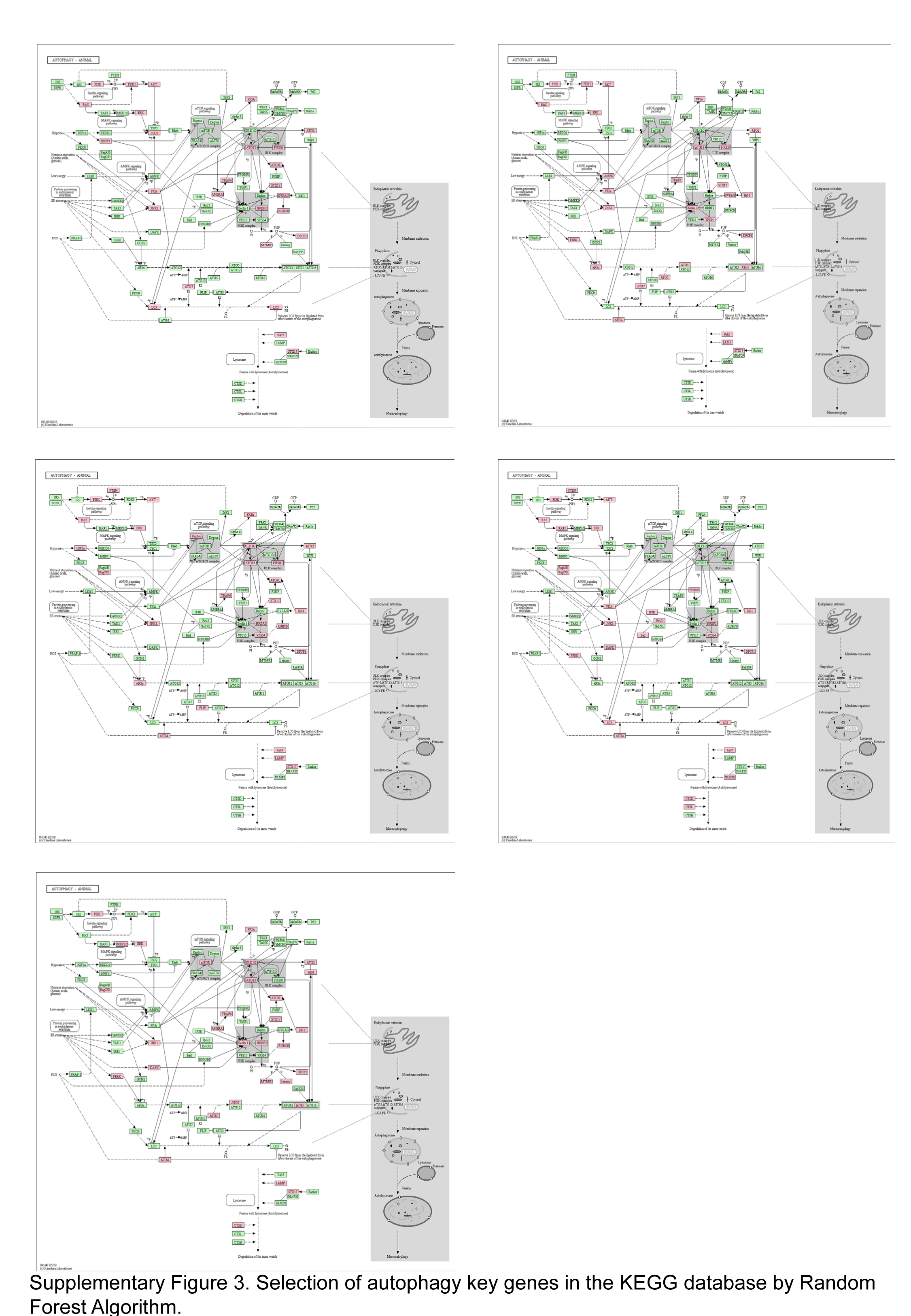

Supplement: Supplementary file 1 [file biomolecules-13-00339-s001.zip › Figure S3.tif]
